# A Novel 3D Osteoblast and Osteocyte Model Revealing Changes in Mineralization and Pro-osteoclastogenic Paracrine Signaling During Estrogen Deficiency

**DOI:** 10.3389/fbioe.2020.00601

**Published:** 2020-06-10

**Authors:** Syeda Masooma Naqvi, Juan Alberto Panadero Pérez, Vatsal Kumar, Anneke S. K. Verbruggen, Laoise M. McNamara

**Affiliations:** Mechanobiology and Medical Device Research Group, Department of Biomedical Engineering, College of Engineering and Informatics, National University of Ireland Galway, Galway, Ireland

**Keywords:** estrogen deficiency, osteoblasts, osteocytes, mineralization, 3D culture, mechanical stimulation

## Abstract

Recent *in vitro* studies have revealed that the mechanobiological responses of osteoblasts and osteocytes are fundamentally impaired during estrogen deficiency. However, these two-dimensional (2D) cell culture studies do not account for *in vivo* biophysical cues. Thus, the objectives of this study are to (1) develop a three-dimensional (3D) osteoblast and osteocyte model integrated into a bioreactor and (2) apply this model to investigate whether estrogen deficiency leads to changes in osteoblast to osteocyte transition, mechanosensation, mineralization, and paracrine signaling associated with bone resorption by osteoclasts. MC3T3-E1s were expanded in media supplemented with estrogen (17β-estradiol). These cells were encapsulated in gelatin-mtgase before culture in (1) continued estrogen (E) or (2) no further estrogen supplementation. Constructs were placed in gas permeable and water impermeable cell culture bags and maintained at 5% CO_2_ and 37°C. These bags were either mechanically stimulated in a custom hydrostatic pressure (HP) bioreactor or maintained under static conditions (control). We report that osteocyte differentiation, characterized by the presence of dendrites and staining for osteocyte marker dentin matrix acidic phosphoprotein 1 (DMP1), was significantly greater under estrogen withdrawal (EW) compared to under continuous estrogen treatment (day 21). Mineralization [bone sialoprotein (*BSP*), osteopontin (*OPN*), alkaline phosphatase (ALP), calcium] and gene expression associated with paracrine signaling for osteoclastogenesis [receptor activator of nuclear factor kappa-β ligand (*RANKL*)/osteoprotegerin *OPG* ratio] were significantly increased in estrogen deficient and mechanically stimulated cells. Interestingly, BSP and DMP-1 were also increased at day 1 and day 21, respectively, which play a role in regulation of biomineralization. Furthermore, the increase in pro-osteoclastogenic signaling may be explained by altered mechanoresponsiveness of osteoblasts or osteocytes during EW. These findings highlight the impact of estrogen deficiency on bone cell function and provide a novel *in vitro* model to investigate the mechanisms underpinning changes in bone cells after estrogen deficiency.

## Introduction

Osteoporosis is a debilitating bone disease, in which severe bone loss occurs leading to fractures of the hip, wrist, or vertebrae (Balena et al., 1993). The disease is most commonly manifested in postmenopausal women when estrogen production is deficient, and affects approximately 30% of postmenopausal women (Melton et al., 2005). Bisphosphonate drugs target bone resorbing osteoclasts but only reduce osteoporosis fractures by 50% (Randell et al., 2002). However, osteoporosis is not simply a disease of bone loss and recent studies have demonstrated that bone tissue composition is fundamentally altered at the tissue level (Gadeleta et al., 2000; Harris et al., 2000; Zioupos and Aspden, 2000; Efstathiadou et al., 2001; Ciarelli et al., 2003; Mansell and Bailey, 2003; McCreadie et al., 2006; McNamara et al., 2006; Zhu et al., 2008). In particular, changes in bone tissue mineral occur, which are anatomically distinct, do not occur ubiquitously throughout the proximal femur, and may be associated with alterations in the cell mechanical environment (Riggs et al., 2002; McNamara et al., 2005, 2006; Brennan et al., 2011a, b, 2012b, 2014a; Vaughan et al., 2015; Verbruggen et al., 2015, 2016; O’Sullivan et al., 2019; Parle et al., 2020).

Throughout life, maintenance of normal adult bone relies on biophysical stimulation, whereby osteocytes continually receive mechanical cues from daily skeletal loading (Knothe Tate, 2003; Coughlin and Niebur, 2012; Birmingham et al., 2013) and elicit biochemical signaling to osteoblasts and osteoclasts (Klein-Nulend et al., 1996; McAllister, 2000; Nauman et al., 2001; Kapur et al., 2002; Malone et al., 2007; Wang et al., 2008; Birmingham et al., 2012; Li et al., 2012; Lee et al., 2014). Cellular mechanosensors have been identified in osteoblasts and osteocytes, which transduce mechanical signals into the cell through its interactions with the actin cytoskeleton (Ziambaras et al., 1998; Rubin et al., 2006; Malone et al., 2007; Litzenberger et al., 2010; Hoey et al., 2012). Osteocyte cell processes interact with the surrounding extracellular matrix by means of α_v_β_3_ integrins (McNamara et al., 2009; Cabahug-Zuckerman et al., 2018), which connect the matrix to the intracellular cytoskeleton in combination with other proteins in complexes known as focal adhesions (Hughes et al., 1993; Zaidel-Bar et al., 2004). A variety of intracellular signaling cascades (MAP kinase, Rho–ROCK, and Wnt/β-catenin) are activated upon exposure to mechanical stimulation (Rubin et al., 2006; Bonewald and Johnson, 2008; Arnsdorf et al., 2009; Jacobs et al., 2010). Osteocytes produce biochemicals and proteins that activate osteoblasts and osteoclasts to remodel bone (Poole et al., 2005; Robling et al., 2008; Kramer et al., 2010; Lewiecki, 2011; Wijenayaka et al., 2011; Tu et al., 2012; Baron and Kneissel, 2013; Delgado-Calle et al., 2017). In particular, WNT/β-catenin signaling promotes bone formation by osteoblasts through inhibition of sclerostin, a protein produced by osteocytes (Papanicolaou et al., 2009; Wijenayaka et al., 2011; Moustafa et al., 2012; Spatz et al., 2015; Thompson et al., 2015; Hemmatian et al., 2018). Mechanical loading decreases sclerostin production by osteocytes (Holdsworth et al., 2019) enabling bone formation. Osteocytes also regulate osteoclastogenesis and bone resorption through the upregulation or downregulation of receptor activator of NFκB ligand (RANKL) and osteoprotegerin (OPG). RANKL is a ligand for receptor activator of NFκB (RANK) present in the cell membrane of osteoclast precursors. The binding between RANKL and RANK triggers signaling pathways, which promote proliferation and differentiation of mature osteoclasts (Wada et al., 2006; Nakashima et al., 2011), which are responsible for bone resorption. OPG is a soluble glycoprotein that acts as a decoy receptor for RANKL, by binding to RANKL and prevents RANK-RANKL association, thus antagonizing the signaling that promotes osteoclastogenesis (Han et al., 2018).

We previously developed an *in vitro* two-dimensional (2D) postmenopausal model to study changes in bone biology during estrogen deficiency. We reported that estrogen withdrawal (EW) altered the osteoblast actin cytoskeleton, PGE_2_ release and expression of RUNX2, COX2, and OPN genes compared to cells that continuously received estrogen (Brennan et al., 2012a, c, 2014b). However, these and other studies of bone mechanobiology and pathophysiology have largely relied on 2D *in vitro* cell culture and applied mechanical stimulation in the form of flow in fluid shear stress (Alford et al., 2003; Litzenberger et al., 2010; Li et al., 2012; Deepak et al., 2017). Yet such 2D approaches do not represent the complexity of *in vivo* biophysical cues, wherein osteocytes are embedded within a three-dimensional (3D) matrix and are simultaneously influenced by mechanical cues arising due to daily physical activity. Indeed, a study of osteocytes cultured in collagen-coated 3D scaffolds revealed a significant increase in expression of important genes (*SOST*, *RANKL*) compared to the same cells cultured in 2D tissue culture plastic (Spatz et al., 2015). Moreover, osteocyte-selective gene expression (E11/GP38) by differentiated human osteocytes cultured in 3D (with biphasic calcium phosphate particles) was comparable to that of mature human cortical osteocytes, whereas gene expression by human osteoblasts cultured on 2D tissue culture plastic was limited (Boukhechba et al., 2009). Specific matrix properties have been identified that govern osteocyte differentiation and mineralization by osteoblasts *in vitro* (Mullen et al., 2013; Mc Garrigle et al., 2016). In particular, it has been shown that matrix stiffness (0.58 kPa) controls the phenotypic shift from osteoblasts to osteocytes in a 3D environment in terms of osteocyte dendrite formation. Such strategies provide a means of investigating osteocyte biology in a 3D environment.

To more faithfully represent the complexity of bone mechanobiology *in vivo*, a 3D culture and bioreactor approach is required. *In vitro* bioreactors provide a physical growth environment for cells and tissues by subjecting them to various types of mechanical forces, such as hydrostatic pressure (HP) (Klein-Nulend et al., 1986; Pörtner et al., 2005; Chen and Hu, 2006; Henstock et al., 2013; Reinwald et al., 2015). We have previously developed a HP bioreactor that can enhance the *in vitro* mineralization potential of human mesenchymal stem cells (Freeman et al., 2017). HP has further been shown to enhance mineralization and bone formation when applied to osteoblasts, osteocytes, cell−seeded constructs and *ex vivo* cultures of chick femurs (Roelofsen et al., 1995; Liu et al., 2010; Henstock et al., 2013; Reinwald et al., 2015). However, no such 3D bioreactor approach has been applied to investigate the interaction between estrogen deficiency, osteoblast-osteocyte differentiation, and mechanical loading.

In this study, we tested the hypothesis that estrogen deficiency alters mineralization and pro-osteoclastogenic potential of osteocytes in 3D constructs under cyclic loading. Specifically, we (1) developed a simplified 3D osteocyte model by combining matrix-based and bioreactor strategies and (2) applied this model to investigate whether estrogen deficiency leads to changes in osteoblast to osteocyte transition, mechanosensation, mineralization, and paracrine biochemical signaling associated with bone resorption by osteoclasts.

## Materials and Methods

### Osteoblast Cell Expansion and Estrogen Pre-treatment

To closely mimic the *in vivo* pre-menopausal physiological environment, and to accustom the cells to the estrogen environment prior to our estrogen deficiency experiments, we first expanded MC3T3-E1 osteoblastic cells (ATCC, United States) in standard αMEM supplemented with 10% fetal bovine serum (FBS), 100 U/mL penicillin, 100 μg/mL streptomycin, 2 mM L-glutamine, and 1 × 10^–8^ M 17β-estradiol (a naturally occurring estrogen derived from cholesterol) and maintained the cells at 37°C in a humidified 5% CO_2_ environment. This estrogen dosage is within the normal physiological circulating serum levels in mice (Porter et al., 2002; Wood et al., 2007; Haisenleder et al., 2011). Cell culture media was replenished every 3–4 days. The pre-treatment duration (14 days) was determined following preliminary experiments and was deemed to be an appropriate duration to allow cells to become accustomed to estrogen before subsequent withdrawal (Brennan et al., 2014b). All reagents used in this study were from Sigma–Aldrich unless otherwise stated.

### Gelatin-Mtgase Hydrogel Encapsulation

The estrogen pre-treated MC3T3-E1 cells were suspended in sterile microbial transglutaminase (mtgase) solution containing Activa WM (Aginomoto foods) at 4 × 10^6^ cells/mL. This mtgase/cell suspension was then enzymatically crosslinked with an equal volume of 6% gelatin (type A, 175 Bloom) (ratio of 1:1), to yield a final mtgase concentration of 0.3% w/w, a cell density of 2 × 10^6^ cells/mL and a gelatin concentration of 3%. This concentration for gelatin-mtgase was established based on previously published work from our lab that demonstrated a matrix stiffness of 0.58 kPa controls the phenotypic shift from osteoblasts to osteocytes in a 3D environment in terms of osteocyte dendrite formation (Mc Garrigle et al., 2016). To produce rectangular constructs (3 mm × 4 mm × 13 mm), the gelatin-mtgase cell suspension was pipetted into custom made polydimethylsiloxane (PDMS) wells and allowed to cool at 4°C for 6 min.

### Estrogen Deficiency

These estrogen pre-treated MC3T3-E1 cells encapsulated in gelatin were then cultured under the following experimental conditions: (1) standard growth medium with estrogen supplementation (E) (1 × 10^–8^ M 17β-estradiol) or (2) standard growth medium with no estrogen supplementation, to mimic the onset of estrogen deficiency in postmenopausal osteoporosis, referred to as EW. For the estrogen supplemented group, estrogen was supplemented every 3 days for the duration of the HP and static experiments described below.

### Hydrostatic Pressure (HP) Experiments

After 24 h of incubation under the different estrogen conditions [continued estrogen supplementation (E), EW], constructs from each experimental group were transferred from well plates to sterile, heat-sealed, gas permeable, and water impermeable, EVO cell culture bags (Quest Biomedical, West Midlands, United Kingdom) containing 30 mL of their respective media, as described above. Ten constructs were placed within each EVO cell culture bag and air was removed. For mechanical stimulation, the bags were placed in a custom developed HP bioreactor and were subjected to an intermittent HP regime of 270 kPa and a frequency of 1 Hz for 1 h per day, 5 days per week for the duration of the study (21 days). A pressure of 270 kPa replicates the physiological pressures experienced by osteocytes in the lacunar–canalicular network of load-bearing bones (Zhang et al., 1998; Reinwald et al., 2015; Reinwald and El Haj, 2018). This pressure was found to promote bone growth and mineralization in a developmental model of organotypically cultured *ex vivo* chick fetal femurs (Henstock et al., 2013). Static control groups were also investigated for all experimental conditions (E, EW), and were similarly contained in heat-sealed EVO cell culture bags and maintained in an open water bath (37°C) for the same duration as the mechanical stimulation periods. After each loading bout, mechanically stimulated and static bags were transferred to a humidified incubator and maintained at 5% CO_2_ and 37°C. Constructs were assessed at days 1, 7, and 21 by immunofluorescent staining, micro-CT, biochemical analyses, and PCR, as are described further below.

### DNA Assay

In order to assess the effects of EW on cell number, DNA content was measured after 1, 7, and 21 day(s) of culture. At each time point, constructs were washed twice with PBS, frozen, and stored at −80°C. Constructs were digested with 3.88 U/mL papain in 0.1 M sodium acetate, 5 mM L-cysteine-HCl, 0.05 M EDTA, pH 6.0 at 60°C under constant rotation for 18 h. Cell number was evaluated using the Hoechst 33258 DNA assay as previously described (Haugh et al., 2011).

### Immunofluorescent Staining

Constructs from each experimental group were stained for Actin, DAPI, dentin matrix acidic phosphoprotein 1 (DMP1), α_v_β_3_, and vinculin. Constructs were fixed using 4% paraformaldehyde after 1, 7, and 21 day(s) of culture at 4°C overnight. Cells within the constructs were permeabilized with 0.5% Triton-X in PBS for 10 min at 4°C under agitation. After three washing steps in 1% BSA, the constructs were immersed in 1% BSA blocking solution for 1 h under agitation.

For DMP1 staining, constructs were incubated in mouse monoclonal anti-DMP1 antibody (1:100) at 4°C overnight (Clone 8G10.3, Merck) and then treated with a DylightTM 549 conjugate goat anti-mouse secondary antibody (1:100) (Jackson Immunoresearch) for 1 h under agitation at room temperature. Constructs were further counterstained with phalloidin-FITC at 1.25 μg/mL (1:400) to stain the actin cytoskeleton and DAPI dilactate (1:2000) to stain the nucleus. Negative controls were previously performed on identical MC3T3-E1 encapsulated gelatin constructs, by omitting the primary antibody incubation step and it was found that no DMP1 staining was observed (Mc Garrigle et al., 2016). In this study, Day 1 cell encapsulated gelatin constructs acted as negative controls, since there is no expected DMP1 expression at this early time point and negative staining for DMP1 would confirm that there is no aspecific staining due to the gelatin constructs or in cells prior to differentiation.

We performed α_v_β_3_ and vinculin staining to investigate whether the integrin α_v_β_3_, previously shown to play an important role in osteocyte mechanotransduction under EW in 2D (Geoghegan et al., 2019), is also implicated under EW in 3D. For integrin α_v_β_3_ and associated membrane protein vinculin staining, constructs were incubated in mouse anti-vinculin primary antibody (1:100) at 4°C overnight and then incubated in a DylightTM 549 conjugate goat anti-mouse secondary antibody (1:100) (Jackson Immunoresearch) for 1 h under agitation at room temperature. Constructs were incubated in an anti-α_v_β_3_ antibody directly conjugated to Alexa Fluor^®^ 488 (1:100) (Santa Cruz) overnight at 4°C and then incubated in DAPI dilactate (1:200) to stain the nucleus.

To quantify all immunofluorescent staining, Z-stack imaging was carried out using a Fluoview FV1000 confocal laser scanning microscope system (Olympus) at a magnification of either 20x (air) or 60x (oil immersion) with a step size of 5 or 1 μm, respectively. All stacks were obtained at the same intensity setting between groups. NIH ImageJ software and FV10-ASW 2.0 Viewer software were used to analyze the images. The images used for analysis were created by combining the stacks of images at maximum intensity projections.

### Quantification of Dendritic Cells and Intensity of DMP1 and Integrin α_v_β_3_

Confocal images of actin/DAPI stained constructs were used for quantification of dendritic cells. Cell processes were defined as cellular features composed of actin, located at the cell membrane, which extended for a distance of at least 10 μm from the cell body, as previously described (Mullen et al., 2014). Using this method, cell morphologies were classified as follows: (1) “not connected dendritic” cells exhibited a small cell body and long thin cell processes of at least 10 μm, without forming interconnections with neighboring cells within the constructs, (2) “not dendritic” cells exhibited a spherical or cuboidal morphology and no cell processes, and (3) “interconnected dendritic” cells exhibited a small cell body and long thin cell processes of at least 10 μm, forming interconnections with neighboring cells within the constructs. The percentage of each type of cell classification was quantified using NIH ImageJ software particle analysis, combining the stacks of images at maximum intensity projections. Osteocytes are defined as interconnected dendritic cells.

Cell area and overall fluorescence intensity were measured using the images stained. Cell area was measured by thresholding the images to remove background fluorescence and then using the “wand tool” to select the region of interest around each cell. When the software could not detect the cell perimeter, the region of interest was drawn manually with the “freehand tool.” The cell area thresholds were then used to determine the intensity of the staining. All fluorescent intensities were measured using the integrated density of each cell with their corresponding background integrated density subtracted.

### Micro-Computed Tomography (μCT)

Like constructs for immunofluorescence staining, constructs for micro-computed tomography (μCT) were fixed using 4% paraformaldehyde at 4°C overnight. A construct from each static and mechanically stimulated group was analyzed for mineralization using μCT scanning methods (Scanco Medical μCT100) with the following parameters: 9 mm holder, 45 kVp tube voltage, 200 μA intensity, Al filter, 5 μm voxel size, 300 ms integration time, and a frame averaging value of 2. Samples were scanned in air and paper strips were used to minimize movement within the chamber. A density threshold of 370 mg HA/cm^3^ (3964 Hounsfield units) was established to exclude hydrogel material and where only mineralized regions remained. This threshold level is higher than the standard range for isolating mineral (90–120 mg HA/cm^3^) because hydrogel and collagen materials have higher density ranges (Vetsch et al., 2015, 2017; Manju et al., 2018; Raina et al., 2018) (Supplementary Figure S1). μCT scans required a Gaussian filter (0.8 sigma, 1.0 voxel support) to reduce noise. A volume of interest (VOI) of 3.95 mm^3^ was 3D reconstructed using Scanco Medical software (Switzerland) and evaluated for mineralization properties such as bone volume fraction (BV/TV), bone mineral density distribution (BMDD), and full width at half max (FWHM) to assess the quantity and distribution of mineralized material within the constructs (Vetsch et al., 2015; Melke et al., 2016).

### Extracellular ALP Activity

Cell culture medium was sampled for each experimental group and subsequently analyzed for ALP secreted to the media. ALP activity was determined using a colorimetric assay of enzyme activity (SIGMAFAST p-NPP Kit), which uses p-nitrophenyl phosphate (pNPP) (nmol) as a phosphatase substrate, with ALP enzyme as a standard. After 1, 7, and 21 day(s) EVO cell culture bags containing constructs were maintained in an incubator for 2 h after the last bout of mechanical stimulation before media was removed, frozen and stored at −80°C. After thawing, 40 μL of the medium was added to a 96-well plate in triplicate with a 50 μL of pNPP solution. The plate was shielded from direct light at room temperature for one hour. The plate was read at 405 nm in a Synergy HT Multi-mode microplate reader. Relative colorimetric readings were converted to ALP activity by creating a standard ALP curve. ALP production was normalized to DNA content to establish whether changes in mineralization were related to (1) the overall mineral capacity of individual cells or (2) changes in the cell population available to produce that mineral.

### Mineralization

Calcium content within constructs was determined using a Calcium Liquicolour kit (Stanbio Laboratories, Syntec, Ireland) according to the manufacturer’s protocol. After 1, 7, and 21 day(s) of incubation, constructs were washed twice with PBS, frozen, and stored at −80°C. Constructs were then thawed and digested by adding 1 mL of 1 M hydrochloric acid (HCL) to each construct and maintaining the constructs at 60°C under constant rotation for 18 h. 10 μL each of the digested constructs and assay standards was added to a 96-well plate and 200 μL of the working solution was added. The plate was read on a synergy HT Multi-mode microplate reader at an absorbance of 550 nm, as previously described (Freeman et al., 2015).

### Quantitative Real-Time PCR

Total RNA was isolated with a custom CTAB method (Koster et al., 2016). Briefly, each construct was digested with 250 μL of RNAse-free proteinase K solution 5 mg/mL, containing 5 mM CaCl_2_. The digested constructs were mixed with 500 μL of CTAB solution (2% [w/v] Cetyl trimethylammonium bromide, 1.4 M NaCl, 20 mM EDTA, and 100 mM Tris, pH 8) containing 1% (v/v) β-mercaptoethanol. Then 500 μL of chloroform were added to each construct and centrifuged at 14,000 *g* for 2 min at room temperature. The upper/aqueous phase was transferred to a fresh tube and 800 μL of isopropanol (Fisher) were added to precipitate total RNA by centrifugation (14,000 *g*, 15 min, room temperature). The pellet was washed with 600 μL 70% (v/v) ethanol and dissolved in 20 μL low TE buffer (Thermo Fisher Scientific) for 15 min at 65°C. RNA purity and yield were assessed using a spectrophotometer (DS-11 FX, DeNovix), with 260/280 and 260/230 ratios over 1.8 for all constructs. 1 μg of RNA from each construct was transcribed into cDNA using Qiagen Quantinova reverse transcription kits and thermal cycler (5PRIMEG/O2, Prime). Relative gene expression was studied by quantitative real-time polymerase chain reaction (qRT-PCR). The genes of interest included *ALP* (*alpl*- alkaline phosphatase) for mineralization, *RANKL* (*TNFSF11*—Receptor Activator for Nuclear Factor κ B Ligand) and *OPG* (*TNFRSF11B—*osteoprotegerin) for pro-osteoclastogenic response, bone sialoprotein (*BSP*), *OCN* (*bglap—*osteocalcin), and *OPN (spp1—*osteopontin*)* for osteoblast phenotype and *DMP1* a marker of differentiation to osteocyte phenotype. *Rpl13A* was used as reference gene (Table 1). qRT-PCR was carried out with 12.5 ng of cDNA per construct, using Qiagen Quantinova SYBR Green PCR kit and a StepOne Plus PCR machine (Thermo Fisher Scientific). The PCR was conducted with an enzyme activation step at 95°C for 10 min, and then for 40 cycles with the following steps: 10 s extension at 95°C, specific annealing temperatures for each primer pair (Table 1) for 10 s, and extension 72°C for 20 s. Analysis of the results was done using the Pfaffl method (Pfaffl, 2001), and the results are expressed as relative quantitative changes to constructs from day 1 of static culture under E.

**TABLE 1:** List of primers employed for qRT-PCR, including sequences (5′–3′) and annealing temperatures (Tm).

Gene	Forward primer	Reverse primer	Tm (°C)
*BSP*	CGGCGATAGTTCCGAAGAGG	TTTCTGCATCTCCAGCCTTCT	56.3
*OPN*	AGCAAGAAACTCTTCCAAGCAA	GTGAGATTCGTCAGATTCATCCG	55.2
*RANKL*	CCCATCGGGTTCCCATAAAG	AGCAAATGTTGGCGTACAGG	58
*OPG*	GCCACGCAAAAGTGTGGAAT	TTTGGTCCCAGGCAAACTGT	57.5
*OCN*	CCTGAGTCTGACAAAGCCTTCA	GCCGGAGTCTGTTCACTACCTT	59
*DMP1*	AAGCTAGCCCAGAGGGACAGGCAA	TTATCGGCGCCGGTCCCCGTAC	58
*ALP*	ATCTTTGGTCTGGCTCCCATG	TTTCCCGTTCACCGTCCA	57.2
*Rpl13A*	TACCAGAAAGTTTGCTTACCTGGG	TGCCTGTTTCCGTAACCTCAAG	57.3

### Statistical Analysis

Statistical analyses were performed using GraphPad Prism (version 5) software. Two−way ANOVA was used for analysis of variance with Bonferroni’s *post hoc* tests to compare between groups. The results are displayed as mean ± standard deviation. Significance was accepted at a level of *p* ≤ 0.05. The entire experiment was repeated three times with three to four replicates analyzed for each experimental group per repeat.

## Results

### 3D Osteocyte Model Under Continued Estrogen Treatment

**FIGURE 1:**
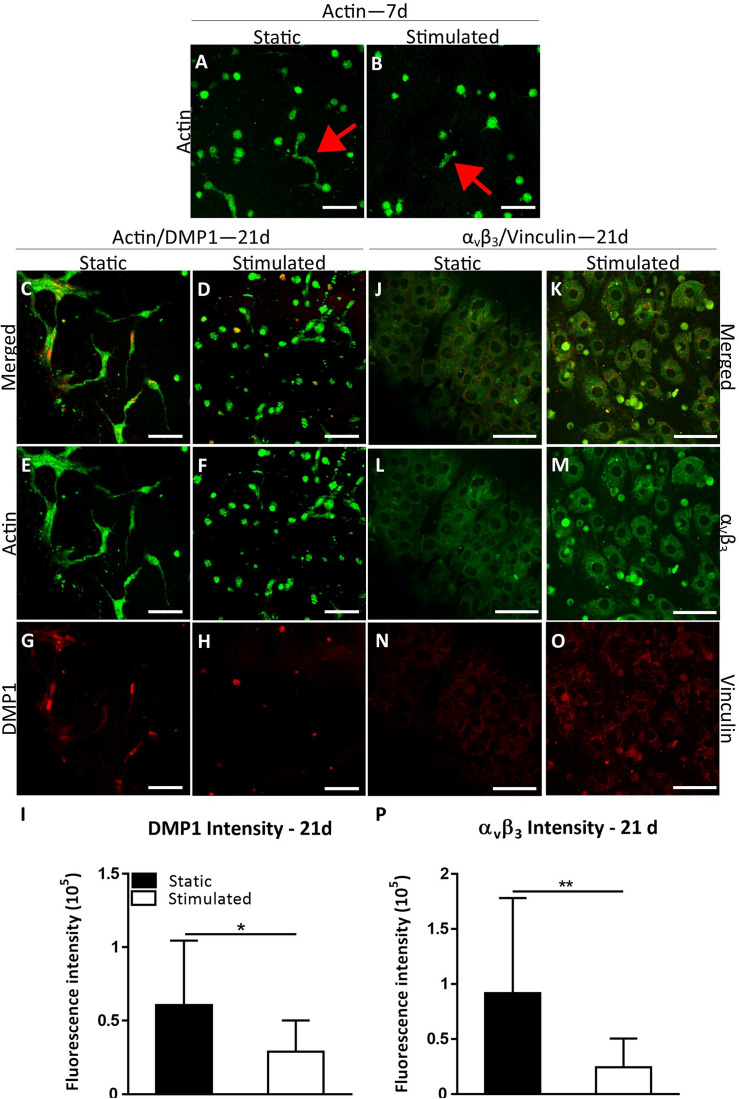
Constructs under continued estrogen treatment under static conditions and mechanical stimulation. Actin (Green) staining at day 7 **(A,B)**. Red arrows indicate dendritic cells. Actin (green)/DMP1 (red) staining at day 21 **(C–H)**. Merged channels for Actin/DMP1 staining **(C,D)**. Green channel for Actin staining only **(E,F)**. Red channel for DMP1 staining only **(G,H)**. DMP1 intensity **(I)**. α_v_β_3_ (green)/vinculin (red) staining at day 21 **(J–O)**. Merged channels for α_v_β_3_/vinculin staining **(J,K)**. Green channel for α_v_β_3_ staining only **(L,M)**. Red channel for vinculin staining only **(N,O)**. α_v_β_3_ intensity **(P)**. Images were taken from the construct surface to a depth of approximately 65 μm. Scale bar = 50 μm. *p < 0.05, **p < 0.01.

We first sought to establish whether this model may be considered an appropriate 3D osteocyte model under normal estrogen conditions (E) and compared static and mechanically stimulated constructs. Actin staining revealed that the MC3T3-E1 cells began to form dendritic cell processes by day 7 under both static and mechanically stimulated conditions (Figures 1A,B). By day 21, interconnected dendritic cells were present (Figures 1C–F). Simultaneously, DMP1 staining was positive at day 21 (Figures 1C,D,G,H) indicative of phenotypic differentiation toward osteocytes. Interestingly, the intensity of DMP1 staining decreased significantly with mechanical stimulation (*p* < 0.05) (Figure 1I). Day 1 MC3T3 encapsulated gelatin constructs stained negatively for the DMP1 marker, which confirms that there was no aspecific staining due to the gelatin constructs or in cells prior to differentiation (Supplementary Figure S2). Next, we performed immunostaining for integrin α_v_β_3_ on day 21 constructs. All constructs stained positively for integrin α_v_β_3_ and vinculin (Figures 1J–O). Semi-quantitative analysis revealed a significant decrease in α_v_β_3_ intensity under mechanical stimulation compared to static culture (*p* < 0.005, Figure 1P).

**FIGURE 2:**
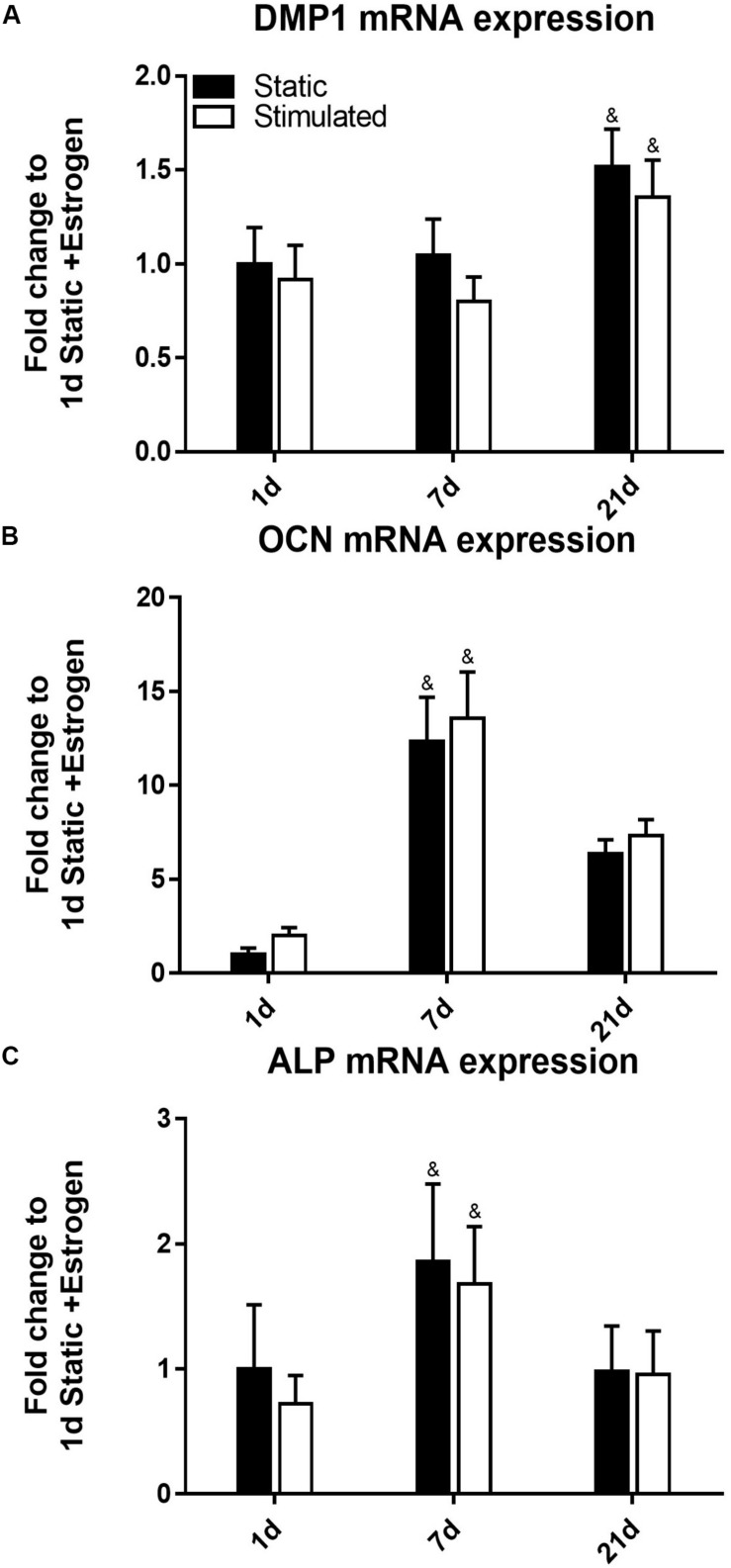
Constructs under continued estrogen treatment under static conditions and mechanical stimulation. Gene expression of *DMP1* **(A)**, *OCN* **(B)**, and *ALP* **(C)**. All constructs normalized to static constructs at day 1, ^&^significance compared to earlier/later time point(s) for the same experimental group; *p* < 0.005.

The time of cell culture had a significant effect on gene expression under all the conditions. For example, the expression of *DMP1* (Figure 2A) was upregulated at day 21 (*p*-value < 0.0005) compared to earlier time points of days 7 and 1. Our *DMP1* gene expression data correlates with the DMP1 staining, which revealed positive staining at day 21 only. Interestingly, the difference in DMP1 intensity observed between static and mechanically stimulated conditions was not apparent in gene expression data. Expressions of *OCN* (Figure 2B) and *ALP* (Figure 2C) peaked at day 7 while by day 21, they were downregulated.

**FIGURE 3:**
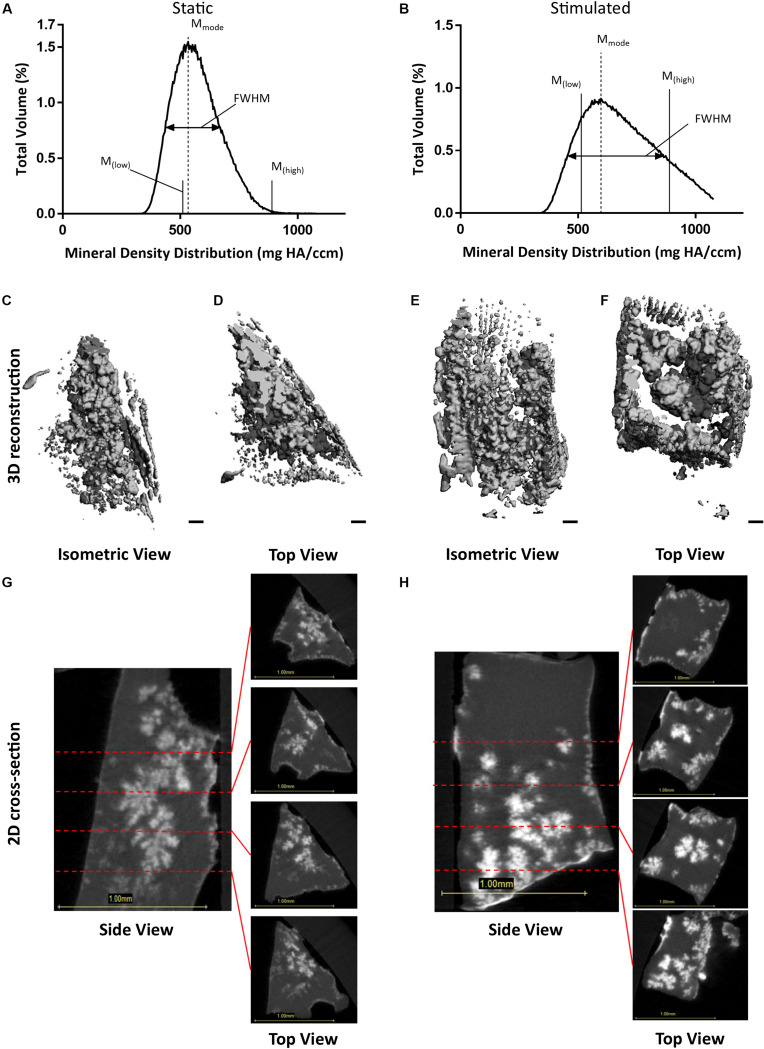
Constructs under continued estrogen treatment under static conditions and mechanical stimulation. Graphs representing BMDD analysis, featuring full width half max bars (FWHM) to quantify heterogeneity, the most frequent mineral density (M_mode_), and markers indicating mineral density at the 25th (M_low_) and 75th (M_high_) percentiles **(A,B)**. 3D reconstructions of the VOI **(C–F)**. Orthogonal view **(C,E)**. Top view **(D,F)**. Scale bars = 100 μm. Grayscale views of cross-section of constructs, alongside four sub-images of example slices, 0.3 mm apart, throughout the constructs **(G,H)**. All scale bars = 1.0 mm.

To confirm that we had mineralization as well as osteocyte differentiation, we performed μCT on our 3D models. Analysis of constructs at day 21 confirmed the presence of clustered regions of density values at and above levels assigned to bone mineral (Vetsch et al., 2015). Figures 3A,B present BMDD analysis of these constructs with corresponding results in Table 2. BV/TV reveal the mechanically stimulated E construct contains ∼4.61% bone-like mineral. The percentage volumes of high mineral density (above the 75th percentile marker) are approximately 16% in the static construct but lower in the mechanically stimulated group (∼13%). Consequentially, the low mineral density (below the 25th percentile marker) is lesser in the static (∼6%), compared to the mechanically stimulated (∼15%) constructs. However, the volume of most frequent mineral value (M_mode_) was highest for the mechanically stimulated E sample (597.55 mg HA/ccm), and lowest for its static counterpart (533.6 mg HA/ccm), indicating the highest density recorded occupies a larger volume of the 3D construct following mechanical stimulation. FWHM, an indicator of mineral heterogeneity, is greater in the mechanically stimulated constructs than the static constructs. 3D reconstructions of the thresholded regions (Figures 3C–F) demonstrate large spaces between mineral clusters. Figures 3G,H reflect this observation, where views of the constructs in grayscale cross-sections (distributions at 0.3 mm intervals) display bone-like mineral along the external layer of the construct, and some highly clustered regions are formed within.

**TABLE 2:** BMDD results for static and mechanically stimulated estrogen and estrogen withdrawn samples, including BV/TV and volume percentages of mineral in the lower, medium, and upper quadrants of distributed density.

			Estrogen	
	Estrogen		withdrawal	
		
	Static	Stimulated	Static	Stimulated
BV/TV%	2.1	4.61	0.68	2.31
M_(low)_ mineral volume%	6.349	15.165	5.530	27.483
M_(medium)_ mineral volume%	76.981	70.876	77.93	71.201
M_(high)_ mineral volume%	16.668	13.965	16.530	1.315
M_mode_ density (mg HA/ccm)	533.5984	597.552	577.9381	560.43
M_mode_ volume%	1.5450	0.913	1.1960	1.297
FWHM (mg HA/ccm)	233.45	403.52	277.31	294.01

*Also included are the value for most frequent mineral density (M_*mode*_) and full width density range at half the maximum volume (FWHM).*

### 3D Osteocyte Estrogen Withdrawal Model

**FIGURE 4:**
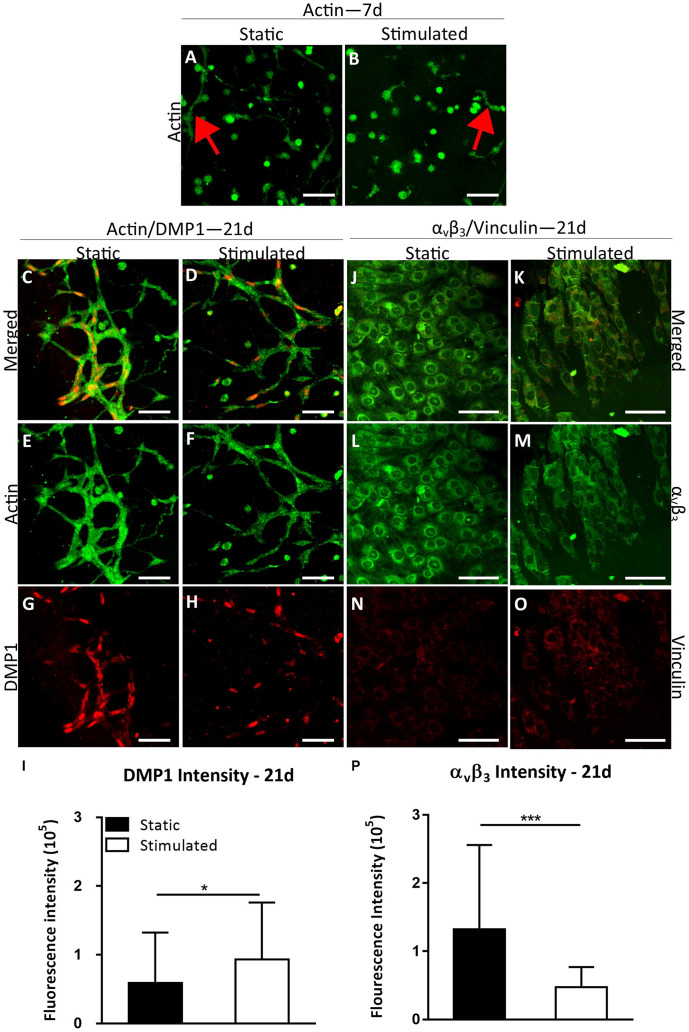
Constructs under estrogen withdrawal (EW) under static conditions and mechanical stimulation. Actin (green) staining at day 7 **(A,B)**. Red arrows indicate dendritic cells. Actin (green)/DMP1 (red) staining at day 21 **(C–H)**. Merged channels for actin/DMP1 staining **(C,D)**. Green channel for actin staining only **(E,F)**. Red channel for DMP1 staining only **(G,H)**. DMP1 intensity **(I)**. α_v_β_3_ (green)/vinculin (red) staining at day 21 **(J–O)**. Merged channels for α_v_β_3_/vinculin staining **(J,K)**. Green channel for α_v_β_3_ staining only **(L,M)**. Red channel for vinculin staining only **(N,O)**. α_v_β_3_ intensity **(P)**. Images were taken from the construct surface to a depth of approximately 65 μm. Scale bar = 50 μm. *p < 0.05, ***p < 0.005.

We next investigated osteocyte differentiation under conditions that mimic the onset of estrogen deficiency in postmenopausal osteoporosis. Cells under EW began to form processes early in the culture period (day 7, Figures 4A,B) and by day 21 most of the dendritic cells were interconnected (Figures 4C–F). Furthermore, DMP1 staining revealed increased DMP1 under EW when compared to E (Figures 4C,D,G,H). Interestingly, after quantification of the mean intensity per cell, the DMP1 intensity increased significantly with mechanical stimulation (*p* < 0.05) compared to static controls, which was contrary to that observed under estrogen conditions (Figure 4I). Furthermore, there was a significant increase in DMP1 intensity under EW (93,458 ± 82,594) compared to E (28,778 ± 21,376) for mechanically stimulated constructs only (*p* < 0.0001). Next, we performed immunostaining of integrin α_v_β_3_ on day 21 constructs. EW constructs stained positively for integrin α_v_β_3_ and vinculin at day 21 (Figures 4J–O), and semi-quantitative analysis revealed that there was a significant decrease in mechanically stimulated groups compared to static groups (*p* < 0.001, Figure 4P). This is similar to results obtained for the 3D osteocyte model under continued estrogen treatment.

**FIGURE 5:**
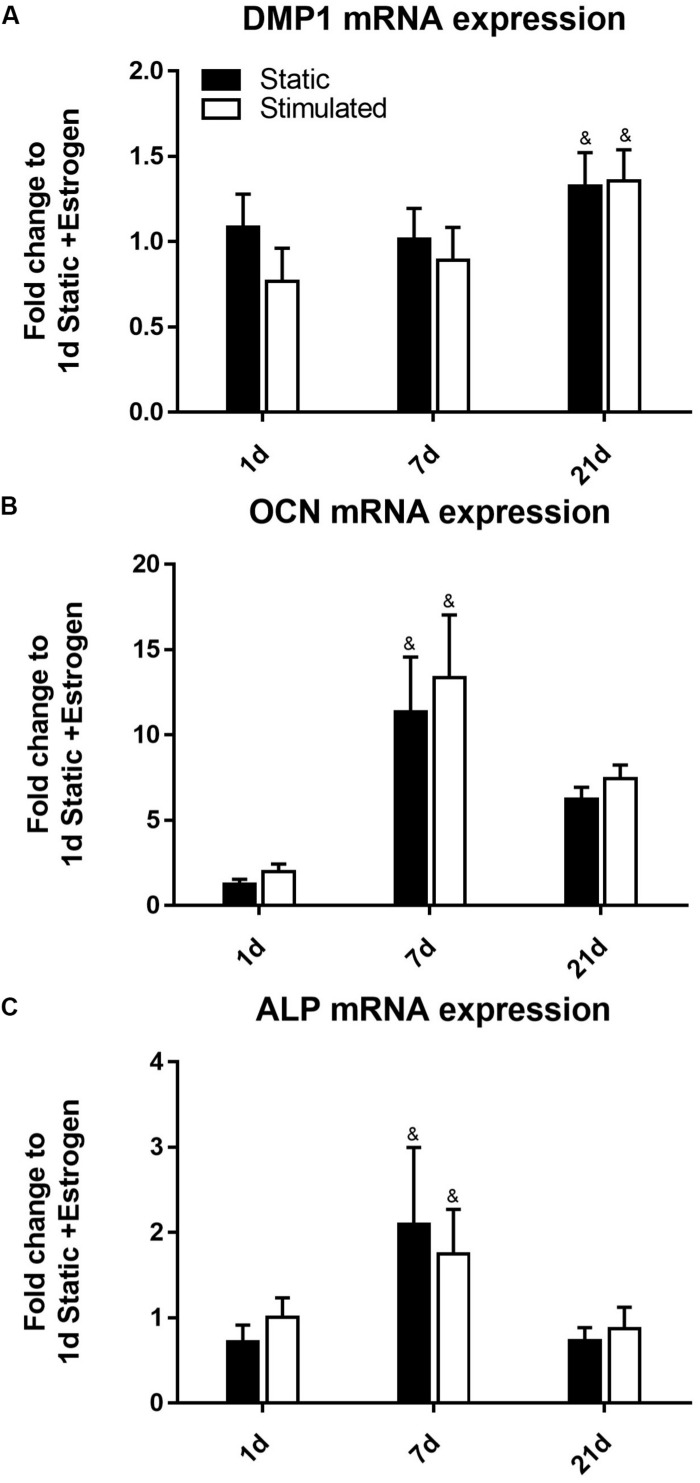
Constructs under estrogen withdrawal (EW) under static conditions and mechanical stimulation. Gene expression of *DMP1* **(A)**, *OCN* **(B)**, and *ALP* **(C)**. All constructs normalized to static constructs at day 1, ^&^significance compared to earlier/later time point(s) for the same experimental group; *p* < 0.005.

*DMP1* gene expression (Figure 5A) was upregulated at day 21 (*p*-value < 0.0005). Our *DMP1* gene expression data correlate with the DMP1 staining, which revealed positive staining at day 21 only. Interestingly, the difference in DMP1 intensity observed between estrogen conditions was not apparent in gene expression data. Expressions of *OCN* (Figure 5B) and *ALP* (Figure 5C) peaked at day 7 while at day 21, they were downregulated.

**FIGURE 6:**
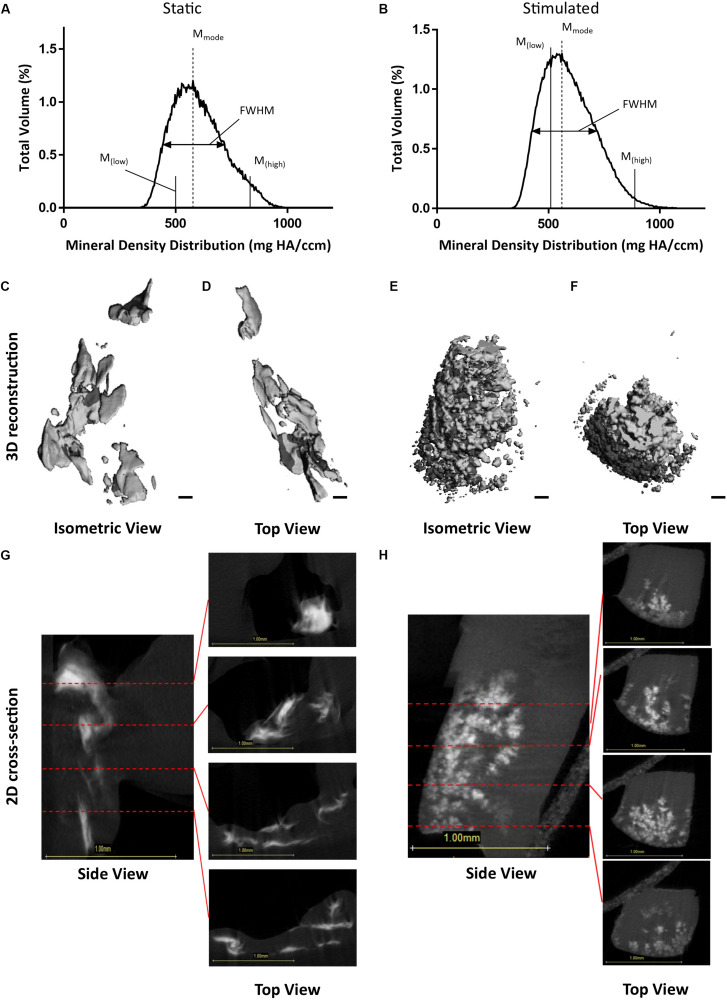
Constructs under estrogen withdrawal (EW) under static conditions and mechanical stimulation. Graphs representing BMDD analysis, featuring full width half max bars (FWHM) to quantify heterogeneity, the most frequent mineral density (M_mode_), and markers indicating mineral density at the 25th (M_low_) and 75th (M_high_) percentiles **(A,B)**. 3D reconstructions of the VOI **(C–F)**. Orthogonal view **(C,E)**. Top view **(D,F)**. Scale bars = 100 μm. Grayscale views of cross-section of constructs, alongside four sub-images of example slices, 0.3 mm apart, throughout the constructs **(G,H)**. All scale bars = 1.0 mm.

Similar to the osteocyte model under continued estrogen treatment, μCT analysis of estrogen deficient constructs at day 21 confirmed the presence of clustered regions of density values at and above levels assigned to bone mineral (Vetsch et al., 2015). Figures 6A,B present BMDD analysis of these four constructs with corresponding results in Table 2. Like the osteocyte model under continued estrogen treatment, the percentage volume of high mineral density is approximately 16% in the static constructs but notably lower in the mechanically stimulated group. Consequentially, the low mineral density range (below the 25th percentile marker) is greater in the static, compared to the mechanically stimulated constructs. In contrast to the osteocyte model under continued estrogen treatment, the volume of most frequent mineral value (M_mode_) was highest for the static EW sample (577.93 mg HA/ccm), and lowest for its mechanically stimulated counterpart (560.43 mg HA/ccm), indicating the highest density recorded occupies a larger space within the 3D construct under static conditions. Like the osteocyte model under continued estrogen treatment, FWHM is greater in the mechanically stimulated constructs than the static constructs. 3D reconstructions of the thresholded regions (Figures 6C–F) demonstrate that the mechanically stimulated constructs formed clustered bone-like mineral regions throughout the cross-sections. Figures 6G,H display these distributions in grayscale cross-sections at 0.3 mm intervals.

### Estrogen Withdrawal Increased Cell Interconnectivity

**FIGURE 7:**
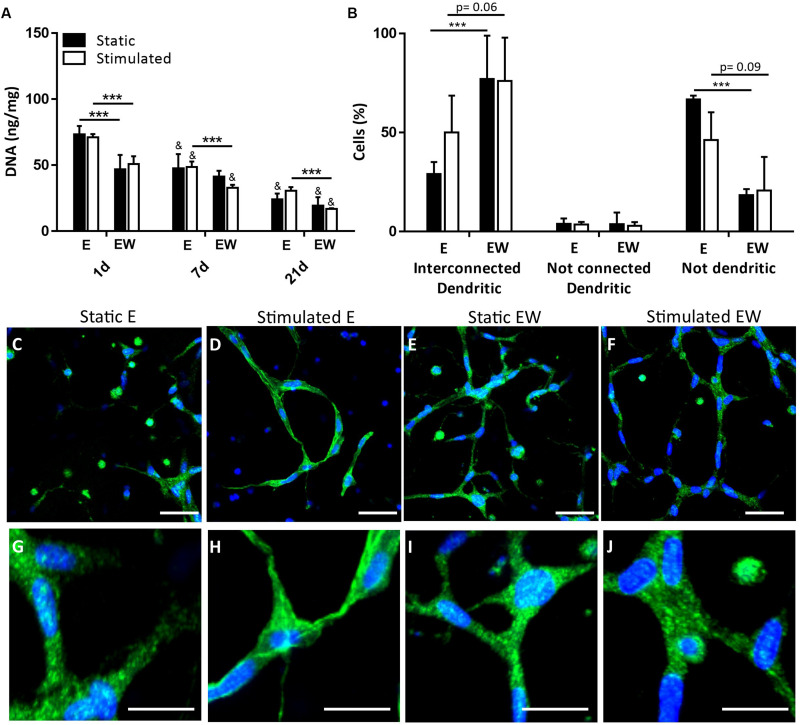
Constructs under both continued estrogen treatment (E) and estrogen withdrawal (EW) under static conditions and mechanical stimulation. Total DNA (ng/mg) content normalized to wet weight at days 1, 7, and 21 **(A)**. Percentage cells after 21 days of culture **(B)**. Actin (green) and Dapi (blue) staining after 21 days of culture illustrating cell morphology **(C–F)**; scale bar = 50 μm. Magnifications of actin/DAPI stained images illustrating interconnections after 21 days of culture **(G–J)**; scale bar = 10 μm. Images were taken from the construct surface to a depth of approximately 65 μm; ***significance compared to E for the same experimental group and time point, ^&^significance compared to earlier time point(s) for the same experimental group, *p* < 0.001.

There was significantly greater DNA content for constructs under E conditions, compared to EW, for both static and mechanically stimulated conditions at day 1. The difference between E and EW was also evident for mechanically stimulated conditions only at day 7 (Figure 7A). DNA content at day 21 was significantly lower than days 1 and 7. The percentage of cells with interconnected processes was significantly higher for the constructs maintained under EW (77.0 ± 21.8%) compared to those maintained under E (29.0 ± 6.1%) for static conditions only (Figure 7B). Under mechanical stimulation, this same trend was observed between EW and E (76.7 ± 21.9 and 50.7 ± 18.6%, respectively), although the difference was not significant. Figures 7C–J illustrate representative actin/DAPI images at day 21, which revealed that interconnected dendritic cells were present under both static and mechanically stimulated conditions. These images were included in the quantification of cell interconnectivity.

### Estrogen Withdrawal Increased Osteoblast Mineralization

**FIGURE 8:**
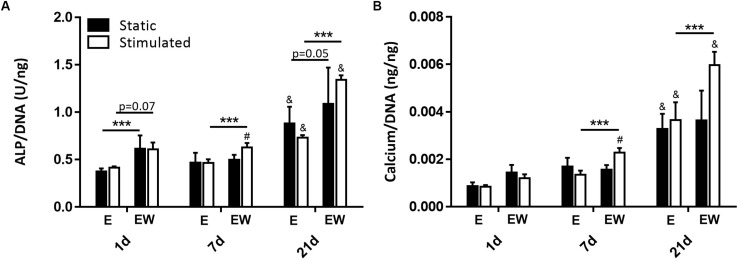
Constructs under both continued estrogen treatment and estrogen withdrawal (EW) under static conditions and mechanical stimulation. ALP activity normalized to DNA at days 1, 7, and 21 **(A)**. Calcium normalized to DNA at days 1, 7, and 21 **(B)**; ^#^significance compared to static for the same experimental group and time point, ^&^significance compared to earlier time point(s) for the same experimental group, ***significance compared to E for the same experimental group and time point; *p* < 0.001.

There was a significant increase in ALP activity under EW compared to E for all time points under mechanical stimulation and for days 1 and 7 under static conditions (Figure 8A). Similarly, there was a significant increase in calcium production for constructs maintained in EW under mechanical stimulation compared to E for all time points (Figure 8B). There were no differences in mineral deposition between static and mechanically stimulated groups for E. However, for EW groups, there was significantly more ALP activity and calcium content under mechanical stimulation at day 7 compared to those maintained under static conditions. Of note, both ALP activity and calcium content increased significantly from days 1 and 7 to day 21. Immunofluorescence of collagen type I illustrated pericellular deposition at day 21 (Supplementary Figure S3) for all groups.

### Estrogen Withdrawal Upregulates Early Expression of Pro-mineralization Markers and Late Expression of RANKL

**FIGURE 9:**
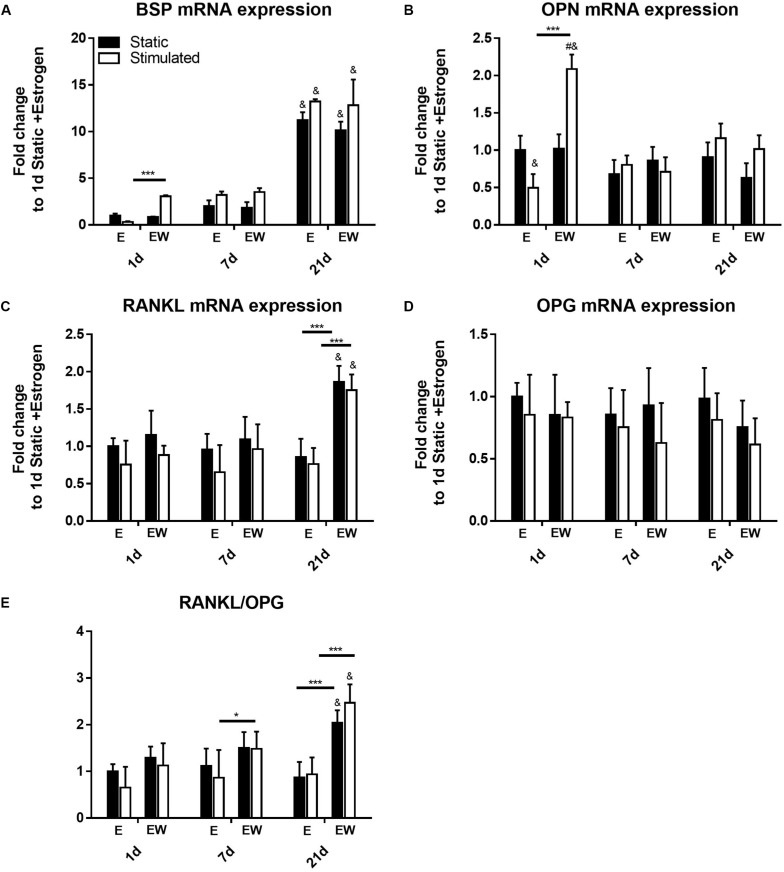
Constructs under both continued estrogen treatment and estrogen withdrawal (EW) under static conditions and mechanical stimulation. Gene expression of *BSP* **(A)**, *OPN* **(B)**, *RANKL* **(C)**, and *OPG* **(D)**. Ratio of expression between *RANKL* and *OPG* **(E)**. All constructs normalized to constructs of day 1 and E, ^#^significance compared to static for the same experimental group and time point, ^&^significance compared to earlier time point(s) for the same experimental group, *p* < 0.05 ***significance compared to E for the same experimental group and time point; *p* < 0.001, *significance compared to E for the same experimental group and time point; *p* < 0.05.

Expression of *BSP* (Figure 9A) was upregulated only in EW under mechanical stimulation at day 1 (*p* < 0.001). By day 21, its expression significantly increased, relative to earlier time points, for all groups regardless of culture condition. Like *BSP* at day 1, expression of *OPN* (Figure 9B) was upregulated in EW under mechanical stimulation (*p* < 0.001). However, at days 7 and 21, its expression decreased to levels comparable to constructs under E.

After 21 days of culture, gene expression of *RANKL* was significantly increased under EW for both static and mechanically stimulated conditions (Figure 9C). *OPG* expression on the other hand was not significantly different among all groups (Figure 9D). Notably, there was a significantly higher *RANKL*/*OPG* ratio (*p* < 0.005) for constructs under EW at day 21 (Figure 9E) for both static and mechanically stimulated conditions (2.04 ± 0.27 and 2.47 ± 0.39 fold increase, respectively).

## Discussion

In this paper, we developed a 3D osteocyte model combining matrix-based and bioreactor strategies to more faithfully represent *in vivo* biomechanical cues. We demonstrated that the strategy successfully enabled osteoblast to osteocyte differentiation, as evidenced by interconnected DMP1 positive dendritic cells that also stained positive for α_v_β_3_ and vinculin. Importantly, we report here for the first time that withdrawal of estrogen altered the osteocyte response compared to continued estrogen supplementation. Specifically osteocytes cultured under EW conditions demonstrated increased DMP1 intensity, cell interconnectivity, mineralization, and paracrine signaling for osteoclastogenesis (*RANKL*/*OPG*). Here, the interaction between mechanical stimulation and EW is of importance, whereby significant changes due to EW were only apparent in the presence of HP, in terms of increases in DMP1 intensity (day 21), ALP activity (days 7 and 21), calcium production (days 7 and 21), expression of markers BSP and OPN (day 1), and ratio of RANKL:OPG (days 7 and 21) under EW.

It must be noted that there are several limitations associated with this study. First, the MC3T3-E1 cell line may not represent the behavior of primary osteoblasts. However, these cells are an accepted, commercially available model of primary osteoblast function, because they express ALP, produce mineral, and can differentiate into osteocyte-like cells *in vitro* (Sudo et al., 1983). Moreover, the use of cell lines eliminates donor variability associated with primary cell culture and thus MC3T3-E1 cells have been studied extensively (Luppen et al., 2003; Balkan et al., 2005; Nakano et al., 2007; Kyono et al., 2012; Wang et al., 2012; Hoac et al., 2013). Second, the withdrawal of estrogen in the current study is abrupt and thus unlike *in vivo* estrogen depletion during menopause in females. However, ovariectomized animal models also undergo an immediate reduction in circulating estrogen levels and these have been shown to be a good representation of osteoporosis (Brennan et al., 2009; Kennedy et al., 2009). Another limitation was that there was only one sample from each group for micro-CT and so our conclusions regarding mineralization are based on the biochemical analysis. Nonetheless, micro-CT data provided a preliminary spatial understanding of mineral deposition, which indicated an increase in low-density mineral and the most frequent mineral density under EW and mechanical stimulation. Our determination of osteocyte-like cells was based on DMP1 positive staining and the presence of dendritic cell processes, following our previous approach (Mullen et al., 2013; Mc Garrigle et al., 2016). However, it should be noted that DMP1 is expressed by both late osteoblasts and osteocytes (Feng et al., 2002) and so these cells may not be mature osteocytes. Future studies could analyze later markers of osteocyte differentiation, in particular the osteocytic marker Sost/sclerostin. Finally, the cell culture media used for these experiments contains phenol red, which has reported estrogenic activity (Berthois et al., 1986; Welshons et al., 1988). However, the concentration of phenol red in αMEM culture media (∼11 mg/L) used in this study is within the range of DMEM/F12 standard culture media (∼9 mg/L), which was shown to be insufficient to cause estrogenic effects (Moreno-Cuevas and Sirbasku, 2000). Moreover, we used the same batch of media for both experimental groups and further supplemented this batch with estradiol (1 × 10^–8^ M) for the estrogen groups only and so we do not have a concern that phenol red confounded the results reported here. Indeed, we anticipate that the use of indicator free media would exacerbate the differences between the EW group and the continued estrogen supplemented group.

This study reported changes in osteoblast-osteocyte transition with 3D encapsulation inducing the differentiation of osteoblast-like cells without the addition of osteogenic growth factors. Specifically, under continued estrogen supplementation, cell processes began to form early in the culture period and by end of culture, we observed a largely interconnected network of cells within the 3D construct. This network was characterized by long osteocyte-like interconnected dendrites. Indeed, osteocytes form a complex interconnected dendritic network *in vivo* allowing for communication with their neighbors and with osteoblasts and osteoclasts on bone surfaces (Bonewald, 2011; Divieti Pajevic and Krause, 2019) and thus for regulation of bone formation and bone resorption. Furthermore, it is established that as an osteoblast differentiates into an osteocyte, expression of the osteoblast marker enzyme *ALP* is greatly reduced (Mikuni-Takagaki et al., 1995; Nakano et al., 2004), as well as reduced expression of *OCN* (Bonewald, 2011) and increased expression of *DMP1* (Narayanan et al., 2003; Rios et al., 2005). In this study, gene expression of *ALP* and *OCN* was upregulated early in the culture period and downregulated by the end of culture for all groups. In contrast, *DMP1* was upregulated by the end of culture and immunostaining of DMP1 revealed positive staining also. Of note, the DMP1 staining appeared to be nuclear for both experimental conditions. In addition to its role in the MAP kinase signaling cascade (Kulkarni et al., 2000), DMP1 may function as a transcription factor in the nucleus (Narayanan et al., 2003). One study examined nuclear localization of DMP1 in MC3T3-E1 preosteoblast cells (Siyam et al., 2012) and demonstrated two subpopulations with either nuclear or cytoplasmic localization of DMP1. In addition, we found positive integrin α_v_β_3_ staining by the end of culture. These results suggest the 3D encapsulation is committing the MC3T3 cells to ultimate differentiation into osteocytes. Indeed, it has previously been demonstrated that 3D encapsulation of osteoblast-like cells induces differentiation (Atkins et al., 2009; Boukhechba et al., 2009; Woo et al., 2011; Uchihashi et al., 2013). However, in the current study, we show that osteocyte differentiation and the formation of interconnections are governed by a 3D matrix without the addition of osteogenic growth factors.

Osteoblasts and osteocytes express estrogen receptor α and β (ERα and ERβ), which play a role in regulating both cell survival and mechanosensation (Lee et al., 2003; Saxon et al., 2012; Castillo et al., 2014). In osteoblasts, estrogen has been shown to play a protective role by inhibiting osteoblast apoptosis and increasing its lifespan. Estrogen exerts these effects due to activation of the Src/Shc/ERK signaling pathway and downregulating JNK, which alter the activity of transcription factors such as CREB and c-Jun/cFos (Kousteni et al., 2001, 2003). Supplementation of estrogen in osteoblasts has also been shown to increase Opg expression (Tomkinson et al., 1997, 1998; Emerton et al., 2010; Florencio-Silva et al., 2018) and augment Cox-2 (via β1 integrins and ERs) response to fluid shear stress (Sterck et al., 1998; Bakker et al., 2005, 2006; Voisin and McNamara, 2015; Deepak et al., 2017; Geoghegan et al., 2019), and decrease RANKL and SOST expression (Brennan et al., 2012c, 2014b). Estrogen supplementation has been shown to have a protective role against osteocyte apoptosis (Plotkin et al., 2005; Marathe et al., 2012), increase connexion 43 gap junction expression and mechanosensitivity (Ren et al., 2013), and increase osteogenic signaling by MLO-Y4 osteocytes (Deepak et al., 2017). Conversely, estrogen deficiency alters osteoblast and osteocyte responses (Sterck et al., 1998; Bakker et al., 2005, 2006; Voisin and McNamara, 2015; Deepak et al., 2017; Geoghegan et al., 2019), and an in particular is associated with an increase in osteocyte apoptosis (Brennan et al., 2012c, 2014b). Ovariectomized rodents and osteoporotic humans display significant increases in osteocyte apoptotic markers (Tomkinson et al., 1997, 1998; Emerton et al., 2010; Florencio-Silva et al., 2018).

Importantly, we observed the notable changes in osteoblast-osteocyte differentiation under postmenopausal conditions, stimulated by EW, as evidenced by actin staining, associated quantification of interconnected dendrites and DMP1 staining. Comparing EW to continued estrogen supplementation, we found the number of interconnected cells identified late in the culture period was significantly greater under EW. DMP1 was also identified within the matrix by immunofluorescence late in the culture period, and its intensity was increased under EW and mechanical stimulation, when compared to continued estrogen supplementation. Of note, *DMP1* gene expression was upregulated over time with no significant difference between groups. Like continued estrogen supplementation, we found positive integrin α_v_β_3_ staining by the end of culture. The quantification from fluorescence staining of α_v_β_3_ in this study revealed no significant differences between estrogen conditions. Importantly, our results show for the first time the effects of estrogen deficiency in a 3D construct.

It is interesting that early in the culture period, MC3T3 cell number was lower under EW when compared to cells that received continued estrogen supplementation. This reduction in cell number could be explained by cell death or a reduction in proliferation and concomitant differentiation (Ruijtenberg and van den Heuvel, 2016). Notably, we also observed increased expression of osteogenic markers *BSP* and *OPN* mRNA under mechanical stimulation at early timepoints. Both proteins increase bone cell adhesion in the mineralized collagen matrix during bone formation (McKee and Nanci, 1996; Giachelli and Steitz, 2000; Malaval et al., 2008). *BSP* is produced by osteoblasts, while *OPN* is produced by both osteoblasts and osteoclasts (Denhardt and Noda, 1998; Lenton et al., 2015; Icer and Gezmen-Karadag, 2018). Although *BSP* acts as an initiator of hydroxyapatite crystal formation in the bone matrix, *OPN* has an antagonistic effect and is a strong inhibitor of hydroxyapatite crystal growth (Boskey et al., 2002; Harmey et al., 2006). The upregulation of both of these genes under EW may be a reflection of the increase in both mineralization and bone resorption observed during early osteoporosis (McNamara, 2010). Taken together the early reduced cell number and *BSP* and *OPN* mRNA expression might indicate that under EW the MC3T3s transitioned toward osteocytes as soon as estrogen was withdrawn.

With regard to mineralization, groups maintained under EW resulted in greater ALP activity early in the culture period and this difference was maintained with time in culture. EW caused no effect during the time frame of the study at the transcription level (*ALP* gene expression). Of note, there was a difference in gene expression and protein activity at day 21, with reduced ALP gene expression and increased ALP protein activity compared to day 7. It should be noted that gene expression is transient, and precedes protein expression, and as such our gene expression time points (days 1, 7, and 21) may not have captured the upregulation of ALP gene expression, but our results do reflect ultimate changes in ALP proteins that arise in estrogen deficiency. However, cells maintained under EW demonstrated increased calcium content as evidenced by biochemical analyses. This correlates with our previous 2D *in vitro* study which reported that EW caused significantly increased osteoblast mineralization compared to continued estrogen supplementation (Brennan et al., 2014b). Moreover, higher mineral content has previously been reported in osteoporotic bone compared to healthy bone from human patients (Dickenson et al., 1981). In another study, analogs of gonadotrophin releasing hormone (GnRH) induced short-term estrogen suppression which resulted in bone accumulation with a higher mineralization density (Boyde et al., 1998). *In vivo* studies have shown similar results; estrogen deficiency caused by ovariectomy increased plasma levels of systemic factors that stimulate proliferation and differentiation of osteoblasts in rats and thus increased bone formation compared sham-operated rats (Yokose et al., 1996). In the same study, osteoblasts from the ovariectomized rats produced more ALP and mineralized bone nodules compared to osteoblasts from the sham-operated rats. It is important to note that the increased calcium content observed under EW in our study suggests that EW itself may be able to enhance the deposition of mineral in the extracellular matrix even in the absence of osteogenic growth factors. The increased expression of *BSP* and *OPN* with mechanical loading and EW very early in the culture period, as well as the increased intensity of DMP1 by the end of culture under EW, may provide one explanation for the increase in calcium content. These three phosphoproteins of the SIBLING family have been widely implicated in the regulation of biomineralization due to an abundance of phosphorylated serine and threonine acid-rich domains in their composition (He et al., 2019). These domains can bind to calcium and therefore act as nucleators, inhibitors, anchoring molecules, growth modifiers, or as substrates for mineral deposition. Furthermore, *BSP* and *DMP1* have been shown to promote mineralization (Padovano et al., 2015). Gelatin is the only component of our scaffolds and there is no exogenous source of calcium phosphate, therefore all mineral nucleation must be related to the encapsulated cells. It is possible that mineral formation arises either due to some biological mechanism or nucleation on dead cells/cell debris. As cell number was lower under EW and mechanical stimulation, cell death may be a contributor to the higher mineralization reported in that group. However, these same conditions corresponded to higher gene expression of BSP and OPN at the early time point of day 1, which suggests early activation of biological mineralization processes. Interestingly, preliminary μCT analysis revealed that mineral depositions were thinly formed along the construct periphery and clustered toward the center, a pattern evident of bone matrix in similarly cultured scaffolds with hMSCs in a silk fibroin scaffold (Hagenmüller et al., 2007; Vetsch et al., 2015). Moreover, deposition of mineral was more heterogeneous within mechanically stimulated constructs compared to the static constructs for both estrogen treated and EW conditions, but that withdrawal of estrogen induces greater homogeneity of bone-like mineral compared to continued estrogen supplementation. It should be noted that the micro-CT mineralization results are strictly indicative as no replicas were available for duplicate analysis and therefore could not be statistically analyzed.

**FIGURE 10:**
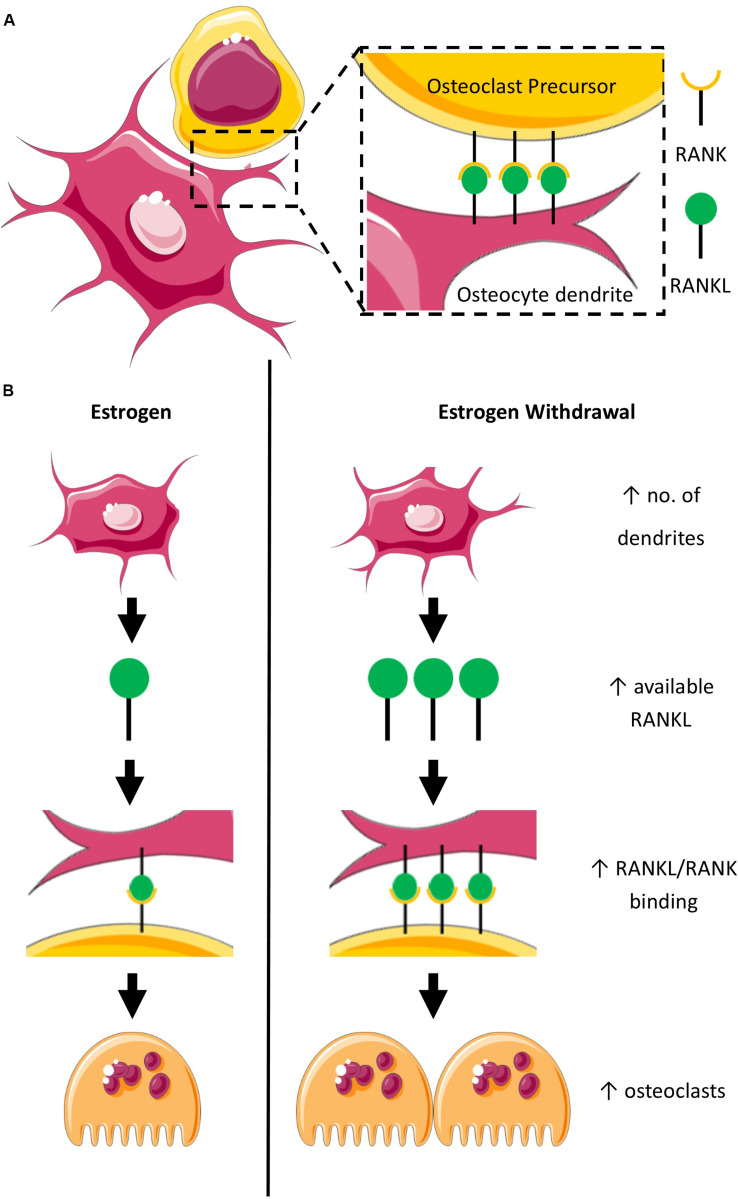
Schematic representation of the proposed mechanism for accelerated osteoclastogenesis in estrogen withdrawal. **(A)** RANK on osteoclast precursor binds with RANKL on dendrites of osteocytes under normal estrogen conditions. **(B)** Estrogen deficiency results in an increase in the number of dendrites which suggests increased expression of RANKL leading to accelerated osteoclastogenesis as a result of interaction of RANKL with RANK on osteoclast precursors.

Here we report an early upregulation of genes *BSP* and *OPN*, which encode proteins important for osteoclast adherence (Ross et al., 1993) and act as substrates for the ECM degrading protein TRAP (Ek-Rylander et al., 1994). We also report a late upregulation of the gene *RANKL*, which encodes the protein RANKL important for bone resorption by osteoclasts (Buckley and Fraser, 2002). These findings are consistent with previously published work in 2D which report exacerbated osteoblast-induced osteoclastogenesis (Allison and McNamara, 2019) and increased RANKL mRNA expression by MLOY4 osteocytes under EW (Geoghegan et al., 2019). During estrogen deficiency, osteoblasts alter paracrine regulation of osteoclast differentiation (TRAP, CTSK, NFATc1) and resorption (Allison and McNamara, 2019). In osteocytes, EW alters integrin-based mechanosensors in MLYO4 osteocyte-like cells, resulting in defective COX2 responses to oscillatory fluid flow, and an increased RANKL/OPG ratio (Geoghegan et al., 2019). Together, these findings suggest that both osteoblasts and osteocytes play a role in enhancing osteoclastogenesis during estrogen deficiency, which may compound the direct effects of estrogen deficiency on osteoclasts. Interestingly, in this study, *RANKL* was upregulated under EW regardless of whether or not the constructs were mechanically stimulated. This is consistent with the increased *RANKL* mRNA expression by osteocytes mentioned earlier, which was demonstrated in static conditions (Geoghegan et al., 2019). Also, we report in this study an increase of dendritic cells under EW in both static and mechanically stimulated conditions. Thus, it is suggested that EW may itself induce these effects. We propose that there may be an increase in available osteocyte membrane-bound RANKL under EW, due to both the increased *RANKL* mRNA expression and increased number of dendrites we have observed in this study. It is established that estrogen regulates bone resorption, in part, by modulating the expression of membrane-bound RANKL on dendrites of osteocytes, which bind with RANK on osteoclast precursors and in this way induce osteoclastogenesis (Figures 10A,B). Based on our results, we hypothesize that EW may enhance osteoclastogenesis as a result of the interaction of RANKL with RANK (Honma et al., 2014) (Figure 10B). Interestingly, it has previously been proposed that estrogen deficiency results in an increase in PGE_2_ production in bone marrow stromal cells, which then induces the expression of RANKL leading to accelerated osteoclastogenesis through interaction with RANK (Kanematsu et al., 2000). Here, we propose that estrogen deficiency results in an increase in number of dendrites, which then would make more RANKL available to bind with RANK and ultimately lead to accelerated osteoclastogenesis.

The current study reveals a link between EW, osteocyte mechanosensitivity, differentiation, mineralization, and expression of pro-osteoclastogenic signaling in osteocyte-like cells embedded in 3D hydrogels subjected to mechanical stimulation without growth factors or mineralization media. Importantly, we report for the first time that osteoblast-osteocyte differentiation increased under estrogen deficiency, as confirmed by actin staining, quantification of interconnected dendrites, and DMP1 staining. These findings highlight the impact of estrogen deficiency on bone cell function and provide a novel *in vitro* osteocyte model to investigate the mechanisms underpinning changes in bone cells after estrogen deficiency.

## Data Availability Statement

The raw data supporting the conclusions of this article will be made available by the authors, without undue reservation, to any qualified researcher.

## Author Contributions

SN and LM conceived the experimental design. SN, JP, and LM drafted the manuscript. SN and JP performed the cell culture. SN and VK performed biochemical analyses, histological analyses, and immunofluorescence. SN and VK performed phase contrast and confocal imaging. JP performed real-time qPCR and quantification of confocal images. AV performed micro-CT scanning. LM supervised the project. All authors read and approved the final manuscript.

## Conflict of Interest

The authors declare that the research was conducted in the absence of any commercial or financial relationships that could be construed as a potential conflict of interest.
