# Supplementary material for: A Novel 3D Osteoblast and Osteocyte Model Revealing Changes in Mineralization and Pro-osteoclastogenic Paracrine Signaling During Estrogen Deficiency
Source: Front Bioeng Biotechnol. 2020 Jun 10;8:601. doi: 10.3389/fbioe.2020.00601 (PMC7326002; doi:10.3389/fbioe.2020.00601)
Supplement: Supplementary file 1 [file Table_1.pdf]

Supplementary Material

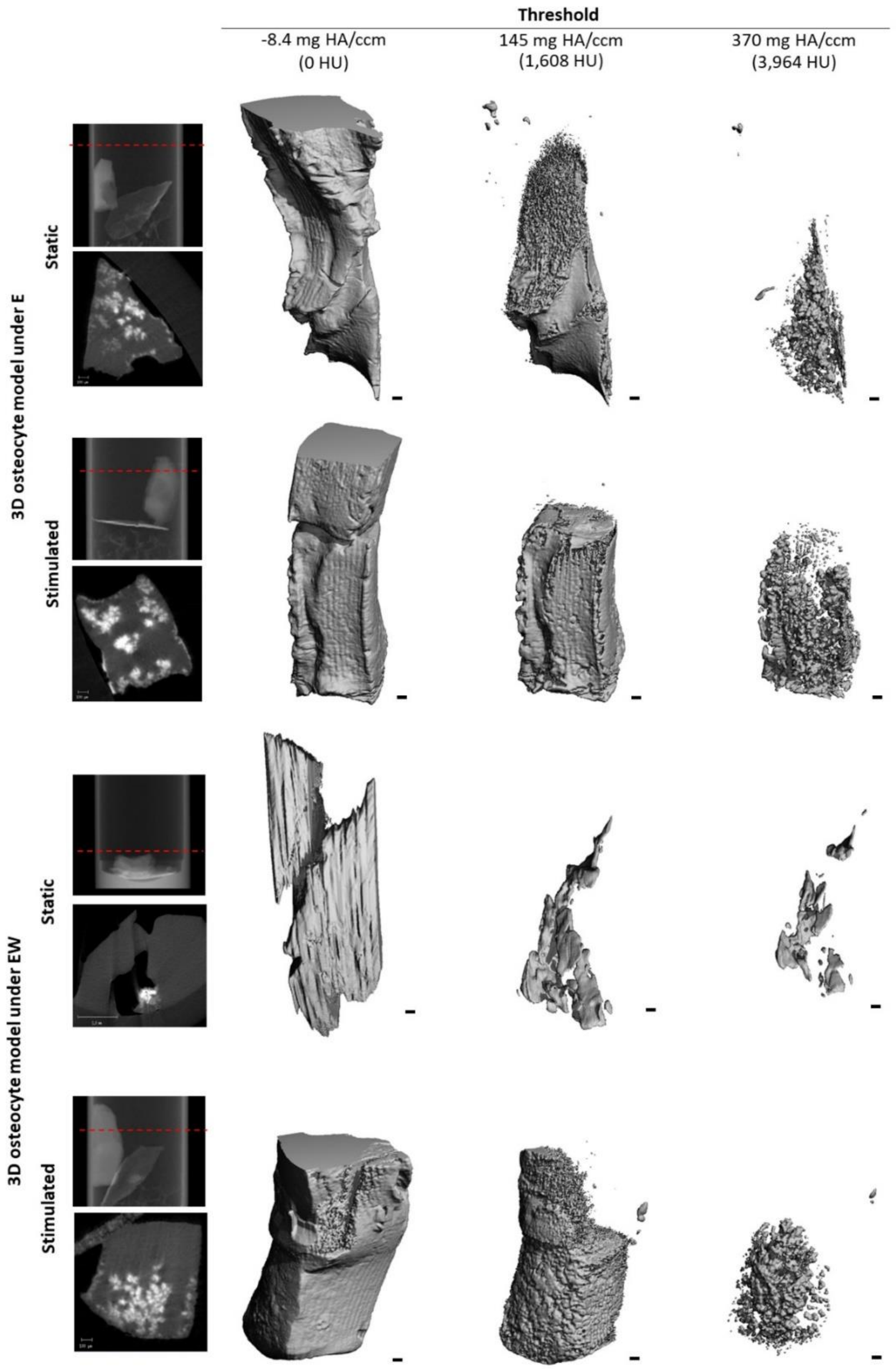

Supplementary Figure 1: µCT scans of constructs at day 21 presented as Greyscale images. Dashed red line indicates where cross-sectional image was taken from. Mineralized regions can be seen in white and hydrogel material as grey. 3D reconstructions of the VOI shown for thresholds -8.4, 145 and 370 mg HA/cm^3. Scale bars = 100 µm.

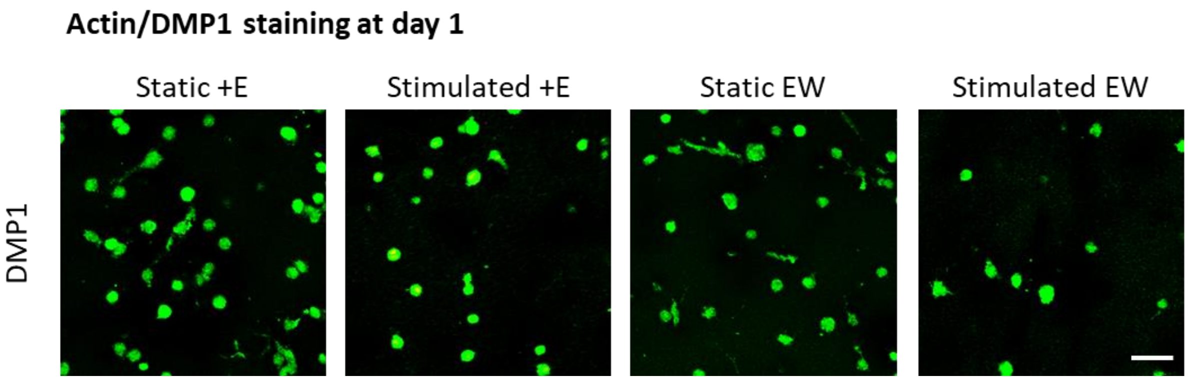

Supplementary Figure 2: Actin (green) and DMP1 (red) immunofluorescence staining of gelatin constructs at Day 1 confirming that there was no aspecific staining for DMP1 (red) due to the gelatin constructs or in cells prior to differentiation; Images were taken from the construct surface to a depth of approximately 65 µm. Scale bar = 50 µm.

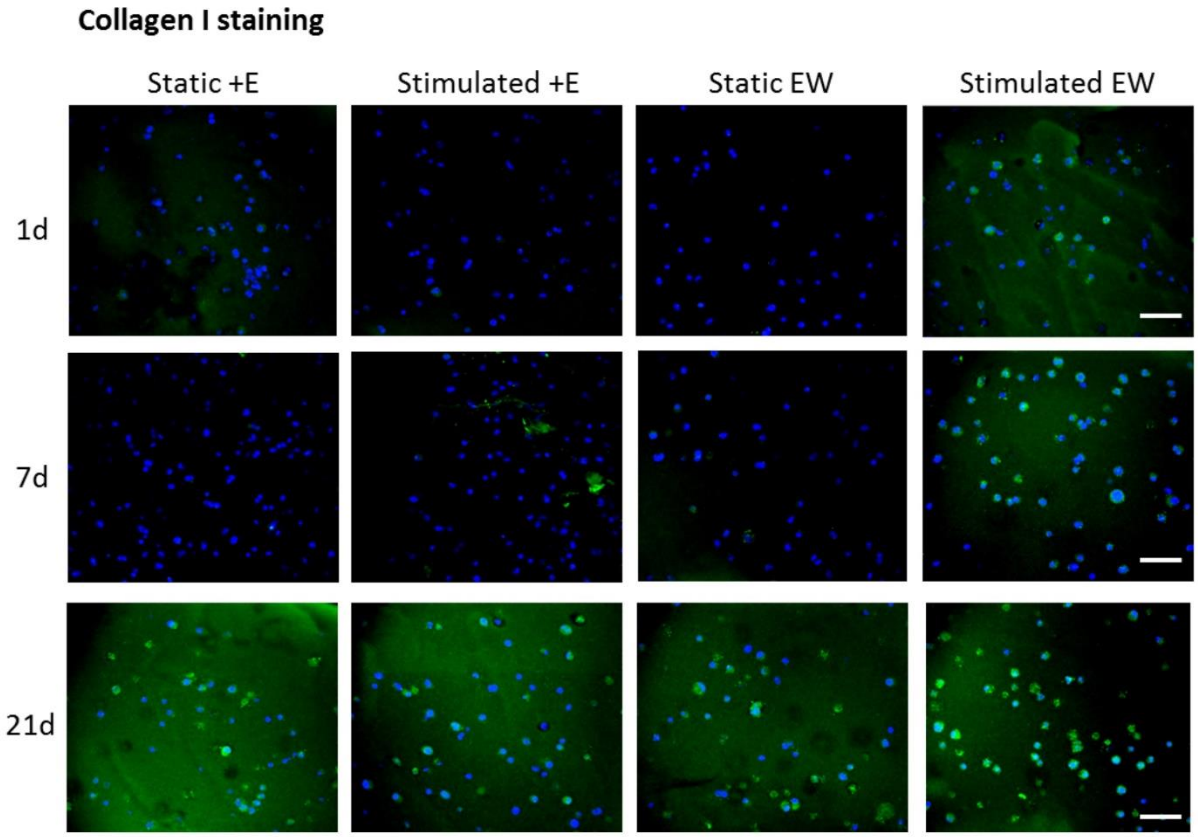

Supplementary Figure 3: Collagen 1 immunofluorescence staining of day 1, 7 and 21 constructs; Images were taken from the construct surface to a depth of approximately 65 µm. Scale bar = 50 µm.
